# Loss of EphA7 Expression in Basal Cell Carcinoma by Hypermethylation of CpG Islands in the Promoter Region

**DOI:** 10.1155/2022/4220786

**Published:** 2022-01-22

**Authors:** Jie Liu, Na Yu, Xiao Feng, Yan He, Kang Lv, Haiping Zhu, Jiandong Wang

**Affiliations:** ^1^Department of Dermatology, Taixing People's Hospital, Jiangsu Taixing 225400, China; ^2^Department of Radiotherapy, Taixing People's Hospital, Jiangsu Taixing 225400, China; ^3^Department of Pathology, Jinling Hospital, Nanjing University School of Medicine, Nanjing 210002, China

## Abstract

Basal cell carcinoma (BCC) is the most common malignancy worldwide, with increasing incidence. BCCs present low mortality but high morbidity, and its pathogenesis remains unclear. Eph receptors have been implicated in tumorigenesis. EphA7 plays a role as a tumor suppressor in certain cancers. We checked EphA7 expression levels and methylation status in a set of BCCs, benign skin diseases, and compound nevus tissue samples using immunohistochemistry. EphA7 protein was positively expressed in normal basal cells, benign skin diseases, and compound nevus cells, but lost in areas of BCC tissues. We detected hypermethylation in BCC tissue samples with reduced expression of EphA7. There is a significant relationship between the expression level of EphA7 receptor protein and the methylation status of CpG islands in the EphA7 promoter region (*P* < 0.001). To our knowledge, this is the first study to report the EphA7 expression profile and hypermethylation of EphA7 in BCC. The role of the EphA7 gene and the status of hypermethylation in tumorigenesis and treatment of BCC warrant further investigation.

## 1. Introduction

According to the American Cancer Society, skin cancer is the most common cancer, accounting for about half of all cancers in the United States [1]. Basal cell carcinoma (BCC) is the most common malignant neoplasm in humans and constitutes approximately 80% of all nonmelanoma skin cancers, with increasing incidence [2]. BCC usually arises from the basal cells of the epidermis of follicular structures, although a small percentage may originate from the outer root sheath of the pilosebaceous unite. BCC is the most common cancer of the skin and has a good prognosis. Despite the low mortality rates and the rare occurrence of metastasis, basal cell carcinoma may be locally invasive and relapse after treatment, causing significant morbidity. This could be due to the existence of cancers stem cells in surgical margins of BCCs, as suggested by Milosevic et al. [3]. Local tissue destruction and disfigurement can be considerable if not limited by early detection and treatment. The etiology of BCC is multifactorial, involving a combination of genotype, phenotype, and environmental factors [2]. UV radiation exposure is the most important environmental risk factor, while other risk factors include childhood sunburns, family history of skin cancer, tanning bed use, chronic immunosuppression, photosensitizing drugs, ionizing radiation, and exposure to carcinogenic chemicals [4]. Although the pathogenesis of BCC is still unclear, many genes are thought to be involved. The patched/hedgehog intracellular signaling pathway is responsible for regulating cell growth and is associated with BCC development [5]. Inactivation of PTCH1 or activating SMOm mutations leads to aberrant hedgehog pathway activation and BCC formation. P53 mutations affecting UV defection are also common in BCCs [6–10].

Erythropoietin-producing human hepatocellular receptor (Eph) is a superfamily of tyrosine kinase receptors, which plays a role in embryonic development and cancers [11–15]. There are two subfamilies of Eph receptors (EphA and B) and two ligands for Eph receptors (EphrinA and B). The Eph/Ephrin proteins are differentially expressed in various adult human tissues and cancers [13, 16–19]. Eph/Ephrin signaling affects cell morphology, migration, and adhesion, all of which play a pivotal role in tissue maintenance [12, 15, 20]. Emerging evidence points to a dual role for Eph receptors in both tumor promotion and suppression [21]. For example, EphA2 is highly expressed in certain human cancers and plays a role as an oncogene [22–27], while EphA7, an Eph receptor, plays roles in the development of the central and peripheral nervous system, limb patterning, and innervation [28–30]. Expression of EphA7 has been detected in some types of human cancer [31–36]. EphA7 is downregulated in colorectal cancer, prostate cancer, and gastric cancer by hypermethylation of its promoter. However, the expression of EphA7 in BCC remains unclear. In this study, we examined the EphA7 expression in a set of BCCs, benign skin diseases, and compound nevus tissue samples. We also investigated the methylation status in CpG islands of the EphA7 promoter region in BCC samples.

## 2. Materials and Methods

### 2.1. Patients and Samples

A cohort of 67 Chinese individuals, including 45 patients with BCC (including 12 superficial, 30 nodular, and 3 infiltrating subtype), 10 patients with benign skin diseases (6 pilocytoma, 3 erythema papules, and 1 pustule), and 12 patients with compound nevus between 2016 and 2020, was studied. The patients were diagnosed with BCC, benign skin disease, and compound nevus based on clinical signs and a biopsy compatible with pathological findings. Formalin-fixed, paraffin-embedded blocks of samples from these patients were retrieved from the Pathology Department archives of Taixing People's Hospital. The ages of the 67 patients ranged between 43 and 96 years, while the patients' mean age was 69.3 years. The protocol used in this study was approved by the Ethics Committee of the Taixing People's Hospital in accordance with the standards of the 1964 Declaration of Helsinki.

### 2.2. Immunohistochemistry

Immunohistochemical staining was performed using Envision Plus system and DAB kit. Briefly, the 4 *μ*m tissue sections were deparaffinized using xylene, dehydrated in an ethanol gradient, and then rehydrated with deionized water. The sections were autoclaved in 1 mM EDTA buffer (pH 8.0) at 120°C for 2 min and cooled to 30°C. The nonspecific sites in the slides were blocked using 10% normal calf serum in phosphate-buffered saline (PBS) for 10 min. Next, an anti-EphA7 polyclonal antibody (Abgent, San Diego, CA, USA) at a 1 : 600 dilution in antibody diluent solution (Zymed, Invitrogen, Carlsbad, CA, USA) was dropped onto the slides and incubated at 4°C overnight. Following incubation, the slides were washed with PBS, stained with 3.3′-diaminobenzidine, and counterstained with hematoxylin. EphA7 expression was assessed as positive when the cytoplasm was stained brown. The immunoreactivity of EphA7 was evaluated independently by two pathologists. Any differences in results were verified by consensus.

### 2.3. DNA Extraction and Bisulfite Treatment

DNA was extracted from formalin-fixed, paraffin-embedded tissues using the QIAmp DNA FFPE tissue kit (Qiagen, Germen) according to the manufacturer's protocol. Briefly, 10 slides with a thickness of 10 *μ*m from tissues were deparaffinized using xylene and dehydrated by gradient ethanol. The concentration and quality of DNA in the elution buffer were determined by measuring the absorbance at 260/280 nm in a spectrophotometer.

Genomic DNA was subjected to bisulfite conversion using the EZ DNA methylation Gold Kit (ZYMO Research, 17062 Murphy Ave, Irvine, CA92614, USA). Briefly, 1 *μ*g genomic DNA in 20 *μ*l was added to 130 *μ*l CT conversion reagent in a PCR tube. Next, the sample tube was placed in a thermal cycler before performing the following steps: 98°C for 10 min, 64°C for 2.5 h, and 4°C for cool. We next added 600 *μ*l of M-biding buffer to a Zymo-Spin IC column and placed the column into a collection tube. The sample was then loaded into the column and mixed by inverting. The sample was centrifuged at full speed for 30s and the flow-through was discarded. Next, we added 100 *μ*l of M-Wash buffer to the column and centrifuged at full speed for 30s. Next, we added 200 *μ*l of M-Desulphonation buffer to the column and let stand at room temperature for 15 min before centrifuging at full speed for 30s. After rinsing with M-Wash buffer twice, we placed the column into a 1.5 ml microcentrifuge tube, added 30 *μ*l of M-Elution buffer to the column matrix, and centrifuged for 30s at full speed to elute the DNA. The DNA was stored at −20°C for later use.

### 2.4. Methylation Detection

The methylation status of CpG islands in the EphA7 promoter region was detected by a direct sequencing method. First, 1 *μ*l bisulfite-treated genomic DNA was amplified in a 30 *μ*l reaction mixture containing 1× buffer, 1 U Takara ExTaq Hotstart Taq (Takara, Dalian, China), 260 *μ*mol/L dNTPs, and 0.3 *μ*mol/L of the bisulfite PCR (BSP) primer sets. The BSP primers were as follows: 5′-TTAGAGTTGGGTTGGAGATTG-3′ (forward) and 5′-CAATAAACACTTCATTAATAACCC-3′ (reverse); the products were 155 bp long. The PCR involved 2 min at 95°C, 40 cycles of 94°C for 30 s, 58°C for 30 s, 72 cycles for 1 min, and finally 10 min at 72°C. The PCR products were subjected to direct sequencing by Sangon Biotech Company (Sangon Biotech, Shanghai, China).

### 2.5. Statistical Analysis

The statistical significance of intergroup differences was analyzed by chi-squared test. All statistical analyses were performed using SPSS software. For all statistical tests, a two-sided *P* value of <0.05 was considered statistically significant.

## 3. Results

### 3.1. Loss of EphA7 Expression in BCC

**Figure 1:**
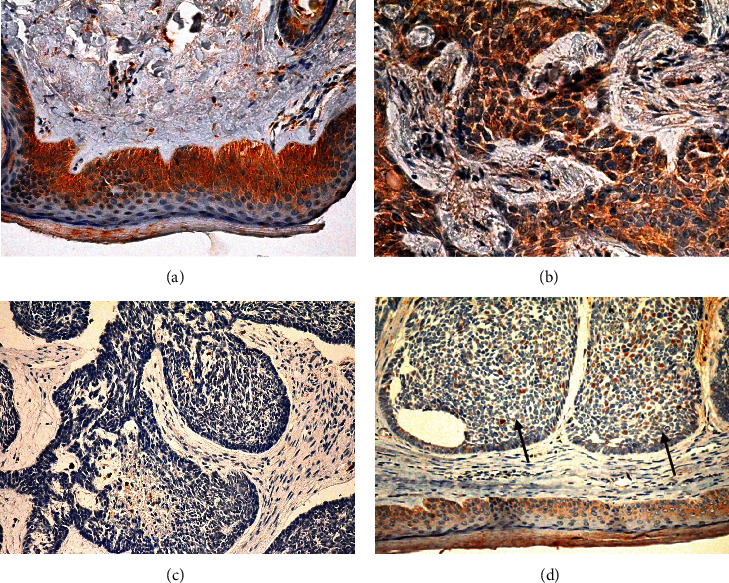
EphA7 expression in BCC was checked using immunohistochemistry. (a) Positive expression of EphA7 in normal basal cells; magnification, 400x. (b) Positive expression of EphA7 in basal cell carcinoma cells; magnification, 400x. (c) Negative expression of EphA7 in basal cell carcinoma cells; magnification, 400x. (d) Loss of expression of EphA7 protein in basal cell carcinoma cells (arrows) compared to that in normal basal cells; magnification, 200x.

**Figure 2:**
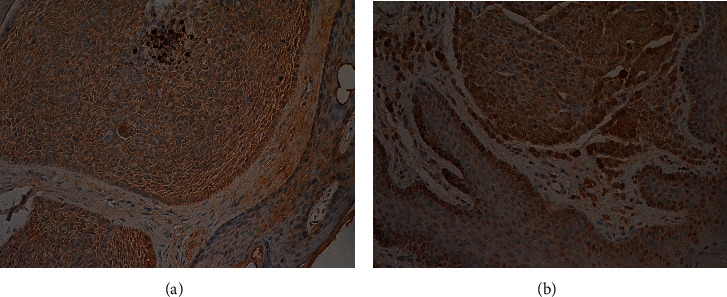
EphA7 receptor was detected in benign skin disease (pilocytoma) (a) and compound nevus (b); magnification, 200x.

The expression level of EphA7 receptor in BCC, benign skin diseases, and compound nevus was checked using a specific anti-EphA7 polyclonal antibody for immunohistochemistry. As shown in Figure 1, EphA7 protein was mainly present in the cytoplasm, as indicated by brown staining, while EphA7 protein was positively detected in normal basal cells (Figure 1(a)). The EphA7 expression level in BCC varied among patients' samples. Positive expression of EphA7 receptor was detected in 25 of 45 cases (55.6%) (Figure 1(b)), while negative expression was noted in 20 of 45 cases (44.4%) (Figure 1(c)). Downregulation of EphA7 was detected in 20 of 45 (44.4%) BCC samples (Figure 1(d)). No significant difference in EphA7 expression was found among different subtypes (*P* = 0.722, Table 1). Positive expression of EphA7 receptor was detected in benign skin diseases (pilocytoma) (Figure 2(a)) and compound nevus (Figure 2(b)).

### 3.2. Hypermethylation of CpG Islands in EphA7 in BCC

**Figure 3:**
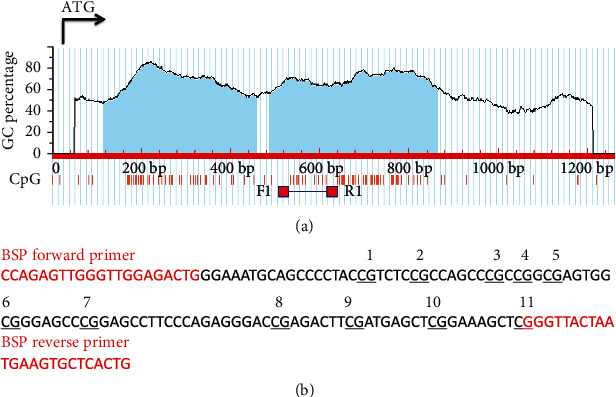
(a). Two CpG islands were identified in the EphA7 promoter region. (b) Bisulfite sequencing primer (BSP) sets, locations, and detailed DNA sequence of amplified fragments.

**Figure 4:**
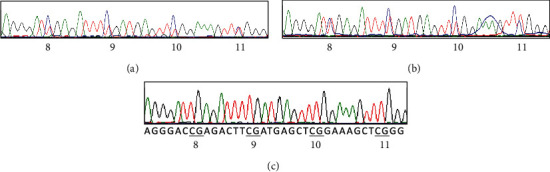
Representative direct sequencing results for hypermethylated (a, b) and unmethylated DNA (c).

Two CpG islands were found upstream of the translation start site ATG in the EphA7 promoter-associated region (Figure 3(a)). The CpG island prediction option is as follows: Obs/Exp (observed/expected CpG ratio) = 0.6, and GC percentage (GC%) = 50. Bisulfite sequencing PCR (BSP) primer sets were selected to amplify bisulfite-treated DNA. There were 11 CG sites in PCR products with 155 bp long (Figure 3(b)). All of the 45 BCC tissue samples were subjected to BSP assay. Hypermethylated CpG islands were detected in 18 of 20 (90%) BCC samples with negative expression of EphA7 and in 1 of 25 (4%) BCC samples with positive expression of EphA7 (Table 2 and Figure 4). There was a significant relationship between the expression level of EphA7 receptor protein and the methylation status of CpG islands in the EphA7 promoter region (*P* < 0.001).

## 4. Discussion

The expression of Eph receptors and Ephrin ligands is often ambiguous in various human cancers. Interestingly, Eph receptors show both tumor promoter and suppressor roles in human cancers. Indeed, EphA7 has been shown to play both tumor-suppressive and oncogenic roles in colorectal cancer, prostate cancer, and lung cancer [31, 37–42]. Upregulation of EphA7 in gallbladder adenocarcinoma and glioblastoma has also been shown to be related to metastasis and poor survival [33, 36]. Moreover, knockdown of EphA7 in lung adenocarcinoma has been found to increase apoptosis through regulation of BAX, Bcl-2, and caspase-3 [43]. These studies showed that EphA7 plays a role as an oncogene through stimulation of migration or invasion and inhibition of apoptosis. However, reduced expression of EphA7 due to hypermethylation of CpG island was found in prostate cancer, gastric cancer, and colorectal cancer [32, 34, 35]. In previous works, we found that downregulation of EphA7 in colorectal cancer and gastric cancer by hypermethylation [34, 35]. Other molecular mechanisms responsible for the loss of EphA7 expression include the long noncoding RNA SNHG14 targeting EZH2 and miR-488 [44, 45].

Recently, the drugs JI-101, XL647, and KB004 have been developed to target various Eph receptors [46–48]. Eph receptors and Ephrin ligands construct a group of complex signaling pathways. It is suitable for the selection of clinical therapy targeting. Akt protein is one of the most common targets downstream of all Eph receptors, and mediates pro- or antitumorigenic effects by regulating proliferation and migration, as seen with the EphA7 receptor [49]. Drugs targeting Eph receptors have made considerable progress owing to the increased insight into the interactions, mechanisms, and expression patterns of Eph receptors. This research direction will create a better understanding of the impact of drugs that intervene with Eph/Ephrin signaling. We believe that more drugs targeting Eph receptors will be evaluated in clinical trials, following which, it is likely that EphA7 receptor will gain a place in the therapy for BCC treatment.

Our data show that EphA7 is differently expressed in BCC tissues. Downregulation of EphA7 was detected in 44.4% (20/45) of samples, while EphA7 expression was absent in 5 superficial, 13 nodular, and 2 infiltrating subtypes. There was no significant difference in EphA7 expression among different subtypes. Hypermethylated CpG islands were detected in 18 of 20 (90%) BCC samples with negative expression of EphA7 and in 1 of 25 (4%) BCC samples with positive expression of EphA7. Two BCC samples had loss of EphA7 expression, with no DNA hypermethylation detected. We deduced that this may be due to other mechanisms such as mutations, microRNA, and long noncoding RNA regulation. However, 4% of BCC samples with positive expression of EphA7 were detected hypermethylation, suggesting heterogeneous expression of EphA7 in BCC samples.

This is a preliminary study for EphA7 receptor expression in BCC. Our results revealed the association of EphA7 downregulation with hypermethylation of CpG islands in the promoter in BCC. However, there are several limitations to this study, including the small sample size, the tissue samples being only from the Asian population with low occurrence rate of BCC, and no functional tests being performed on the EphA7 gene in BCC cell lines, including 5′AzaDC treatment for reverse EphA7 expression of EphA7 in BCC cell lines.

In summary, EphA7 protein was differently expressed in BCC samples but positively expressed in normal basal cells, benign skin diseases, and compound nevus. Reduced expression of EphA7 in BCC is mainly due to hypermethylation of CpG islands. To our knowledge, this is the first time that the EphA7 expression profile and hypermethylation has been reported in BCC. The role of the EphA7 gene and the status of hypermethylation in tumorigenesis and treatment of BCC are worthy of further research.

## Figures and Tables

**Table 1:** EphA7 expression in different subtypes of BCC.

Subtype of BCC	EphA7 (+)	EphA7 (–)	*P* value
Superficial type	7	5	0.722
Nodular type	17	13	
Infiltrating type	1	2	

**Table 2:** EphA7 expression in BCC and association with hypermethylation of CpG island.

	EphA7 (+)	EphA7 (–)	*P* value
Methylated CpG island	1	18	<0.001
Unmethylated CpG island	24	2	

## Data Availability

The data that support the findings of this study are available from the corresponding author, WJD, upon reasonable request.
